# Field Efficacy of Spinetoram for the Management of Coffee Berry Borer (*Hypothenemus hampei*)

**DOI:** 10.3390/insects14030287

**Published:** 2023-03-15

**Authors:** Andrea Kawabata, Roxana Myers, Matthew Miyahira, Nicholas Yamauchi, Stuart T. Nakamoto

**Affiliations:** 1 Department of Tropical Plant and Soil Sciences, College of Tropical Agriculture and Human Resources, University of Hawai‘i at Mānoa, Kealakekua, HI 96750, USA; 2 Daniel K. Inouye Pacific Basin Agricultural Research Center, Agricultural Research Service, United States Department of Agriculture, Hilo, HI 96720, USA; 3 Kona Research Station, College of Tropical Agriculture and Human Resources, University of Hawai‘i at Mānoa, Kealakekua, HI 96750, USA; 4 Department of Human Nutrition, Food, and Animal Sciences, College of Tropical Agriculture and Human Resources, University of Hawai‘i at Mānoa, Honolulu, HI 96822, USA

**Keywords:** *Coffea arabica*, *Beauveria bassiana*, coffee berry borer, Hawaii, integrated pest management, spinetoram

## Abstract

**Simple Summary:**

Coffee is an important agriculture commodity for the State of Hawaii. The cost-efficient and effective management of diseases and pests is essential for the financial stability of an industry facing high labor and production costs. Coffee berry borer, one the most destructive insect pests of coffee worldwide, has recently been introduced to Hawaii coffee farms. This small beetle causes irreversible damage to the coffee seed and severely reduces coffee quality. While an integrated pest management (IPM) approach is in place for the control of this pest, additional management options need to be explored. In this study, the insecticide spinetoram was evaluated in a coffee field in Kona, Hawaii for its efficacy at controlling CBBs and reducing damage to the coffee seed. Spinetoram is derived from a naturally occurring soil bacterium and provides potent, broad-spectrum control of insect pests with low potential for environmental damage. One application of spinetoram caused the mortality of adult CBB, preventing them from moving into the seed and halting reproduction. Spinetoram has potential as a management tool in the CBB IPM program.

**Abstract:**

Coffee berry borer (CBB), *Hypothenemus hampei*, is a damaging insect pest of coffee worldwide. CBB has recently been introduced to Hawaii, so management techniques are still being developed for sustainable and cost-efficient approaches for the effective control of this pest. Field trials were conducted to evaluate the use of spinetoram on CBB infestation and bean damage compared to *Beauveria bassiana* and an untreated control. Initial CBB infestations were similar, and the treatments resulted in no detectable differences in subsequent new infestations. Damage to the coffee beans was reduced by both spinetoram and *B. bassiana* compared to controls as the mortality of adult beetles resulting from the treatments prevented them from moving into the bean (C/D position) from the berry (A/B position). The mortality of adult beetles also prevented reproduction, subsequently reducing future CBB populations in the field. When applied to infested berries, spinetoram reduced live beetle populations in the A/B position by 73% and CBBs in the C/D position by 70% compared to the water control, whereas applications of *B. bassiana* reduced beetles in the C/D position by 37% but had no effect on the live A/B population. An integrated pest management program is recommended for the effective control of CBBs, and the use of spinetoram applications when adult beetles are in the A/B position appears to have potential as another management tool.

## 1. Introduction

Coffee is one of the top agricultural commodities for the State of Hawaii with a farm gate value of USD 61.9 million (cherry basis) for the 2021–2022 season [1]. The cost-efficient and effective management of diseases and pests are essential for the financial stability of an industry facing high labor and production costs. Coffee berry borer (CBB) (*Hypothenemus hampei* Ferrari) is a relatively recent introduction to Hawaii coffee farms with the first discovery in August 2010 in South Kona and the most recent introductions to the Island of Kauai and Lanai in September 2020. This small beetle causes irreversible damage to the coffee bean and severely reduces coffee quality. A devastating pest of coffee worldwide, adult female CBBs bore into the bean and create galleries to lay eggs [2]. Upon hatching, CBB larvae feed on the endosperm, resulting in significant crop loss.

**Figure 1:**
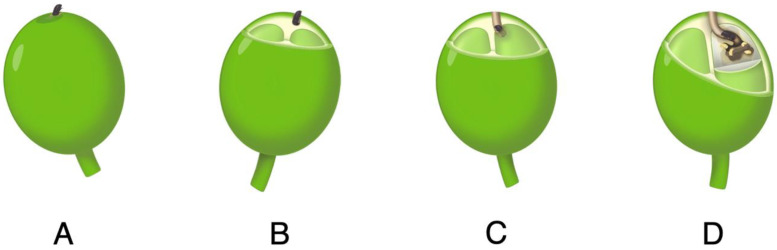
Positioning of the coffee berry borer (CBB) on or in the coffee berry [3]. Position (**A**)—the adult beetle has landed on the berry and initiated a tunneling site. Position (**B**)—the beetle has entered the epidermis of the berry but has not yet infested the bean. Position (**C**)—the beetle has penetrated the parchment and infiltrated the coffee bean. Position (**D**)—the adult beetle has laid eggs and the resulting larvae feed on the endosperm.

In Hawaii, CBB is controlled using an integrated pest management (IPM) approach introduced by Kawabata et al. [3]. CBB IPM includes field sanitation, monitoring, and the use of a biopesticide and chemical pesticides approved by the Hawaii Department of Agriculture. CBB IPM recommendations and an economic model [4] for Hawaii stress the importance of starting the coffee season with as low a CBB infestation as possible and maintaining the control of CBBs with the recommended practices for optimal net benefit. The best management guidance is based primarily on the coffee crop cycle and the lifecycle of CBB and its positioning in the berry [3]. In the A and B positions, infestation has occurred, but the beans are yet undamaged (Figure 1). In the C and D positions, the beans are damaged by the boring beetle and the presence of a new generation of CBBs may be detected. Once in the bean, CBBs are relatively safe from pesticides. The lifespan of a female CBB is approximately 157 days [5] with egg laying taking place for up to 20 days with 2–3 eggs laid per day. As such, it is important to kill CBBs in the A and B positions prior to egg laying and bean damage.

In Hawaii, the entomopathogenic fungi *Beauveria bassiana* is commonly used as a management tool in controlling CBBs in coffee farms. When spores come in contact with the insect’s cuticle, they germinate and colonize the insect causing mortality within 10 days [3] While the proper timing of *B. bassiana* applications is a cornerstone of the IPM approach, other alternatives need to be explored. Many of the chemical insecticides, e.g., endosulfan, used for CBB management in other coffee growing regions are not approved for use on coffee in the U.S. Of six insecticides tested in laboratory bioassays that are approved for coffee applications in Hawaii, only pyrethrins + PBO (Pyronyl™ Crop Spray) demonstrated the direct-contact control of CBBs, and imidacloprid (Admire^®^ Pro) showed moderate indirect-contact CBB control [3].

Spinosyns are naturally derived insecticides resulting from the fermentation of soil bacteria in the genus *Saccharopolyspora* [6]. One component to their mode of action is the disruption of nicotinic acetylcholine receptors. Spinosyns show a potent, broad-spectrum control of insect pests with a reduced risk to non-target organisms. Spinetoram is a partially synthesized spinosyn with greater stability and effectiveness than spinosad while still maintaining its low potential for environmental harm.

The Delegate^®^ WG (water-dispersible granules, 25% spinetoram active ingredient) label [7] claims the control or suppression of foliage feeding insect pests including lepidopterous larvae, thrips, leafminers, and psyllids with minimal disruption of natural enemies. Spinetoram exhibited the highest toxicity against Old World bollworm (*Helicoverpa armigera*) and corn earworm (*H. zea*) compared to three other chemical insecticides in a laboratory bioassay [8]. One application of spinetoram in an almond orchard (*Prunus amygdalus*) reduced populations of peach twig borer (*Anarsia lineatella*) by 98% [9]. Control of Coleopterans has also been demonstrated. The Canadian specimen label of Delegate™ Insecticide, also 25% spinetoram, claims the control or suppression of Colorado potato beetle (*Leptinotarsa decemlineata*), blueberry flea beetle (*Altica sylvia*), and asparagus beetle (*Crioceris asparagi*) [10]. When evaluated as a grain protectant, spinetoram caused mortality and reduced the reproduction of six stored grain pests in the Coleoptera family [11]. Laboratory research found that CBBs could be controlled with Delegate^®^ WG applied at 7 oz per acre (490.4 g per ha) [12]. Replicated trials on CBBs resulted in excellent (100%) direct control and some indirect control.

Field research on the effectiveness of spinetoram is critical for identifying the best-use practices and recommendations for this potential new tool to the current IPM program. The objective of this study was to evaluate the direct and indirect efficacy of field applications of spinetoram in preventing CBB infestation and damage to the coffee bean.

## 2. Materials and Methods

### 2.1. Field Site

A field trial was conducted at an established orchard of *Coffea arabica* cv. ‘Kona Typica’ at the University of Hawaii, College of Tropical Agriculture and Human Resources, Kona Research Station (KRS) in Kealakekua, Hawaii during the 2018–2019 coffee season (harvest from August 2018 through to January 2019). The trees were cultivated primarily in full sun without irrigation. The research station is centrally located within the Kona Coffee Belt and situated at an elevation of 1540 feet (470 m) with coordinates of 19.53, −155.93. Current IPM recommendations were implemented in this field prior to the commencement of this study.

### 2.2. Determining CBB Infestation and Position Pre-Treatment

On 17 May 2018, thirty random branches on at least five trees, were tagged and labeled for each treatment. Prior to the treatment applications, a thirty-branch sampling for each plot was also conducted on nearby non-tagged branches to determine the initial infestation rate and positions of CBB within the berry and bean. Sampling and dissection protocols were followed according to the 2016 Thirty Trees Sampling Method for CBB Monitoring [3].

**Figure 2:**
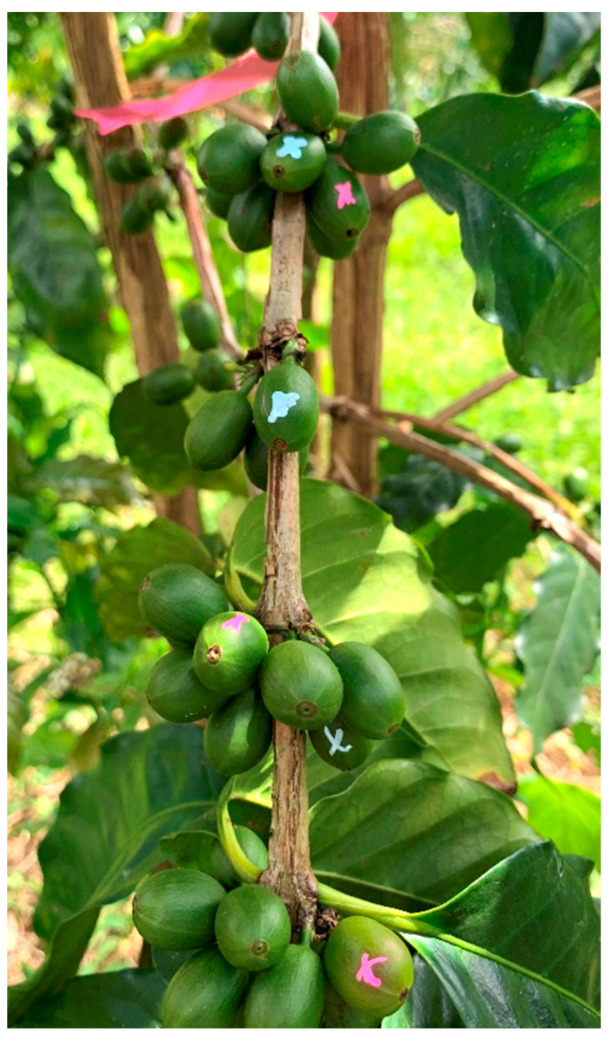
Coffee berries were marked with oil-based pens of different colors to identify initial and new infestations by coffee berry borers during the trial. Marks were placed away from the entry holes.

After pre-sampling, the total green berries and infested berries were counted on each tagged branch and recorded. Berries smaller than 7.0 mm in diameter were not included. Regardless of beetle presence, berries with an entrance hole made by CBBs, typically near the flower scar, were counted as infested. All infested berries were marked with a DecoColor™ opaque paint marker, as shown in Figure 2.

### 2.3. Experimental Design

**Figure 3:**
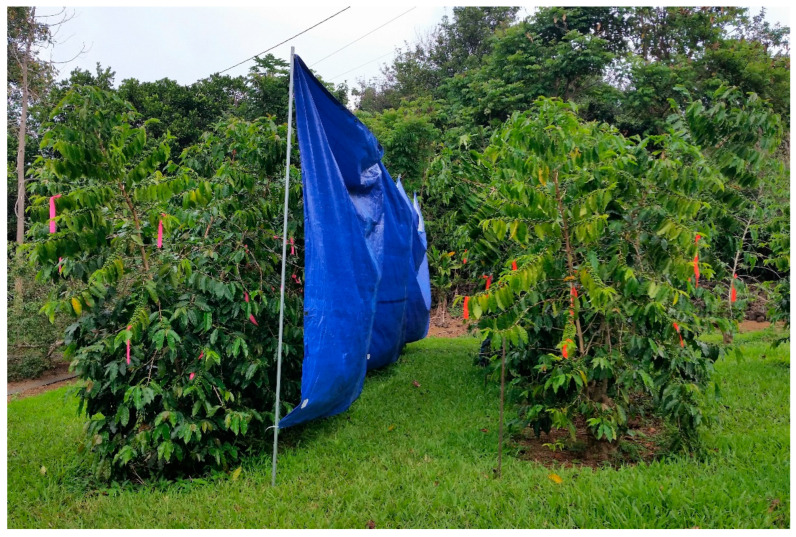
A physical barrier was erected between treatment plots to avoid drift and cross-contamination. These barriers were removed following the spray applications.

On 18 May 2018, the day of treatment (DOT), physical barriers (Figure 3) were erected between rows to prevent drift during treatment applications. A three-gallon hand-held pump sprayer was used to apply the treatments. The three treatments included (1) water (negative control), (2) spinetoram (Delegate^®^ WG, Dow AgroSciences, Indianapolis, IN, USA) at a rate of 5 oz per acre (350.3 g per ha), and (3) *B. bassiana* (BotaniGard^®^ ES, BioWorks, Victor, NY, USA) at a rate of 32 oz per acre (2338.5 mL per ha) plus Widespread Max (Loveland Products, Loveland, CO, USA) at a rate of 0.1% *v*/*v*. Widespread Max is an organo-silicone adjuvant commonly combined with *B. bassiana* for improved coverage. The water rate per acre was determined to be 50 gallons. Branches, foliage, and berries were sprayed to wetness and excessive runoff was avoided. The spray treatments of water and spinetoram were conducted that morning and *B. bassiana* was sprayed in the afternoon according to CBB IPM recommendations [3].

### 2.4. Determining CBB Infestation and Position Post-Treatment

New CBB infestations were noted 7 and 14 days after treatment (DAT). At 7-DAT, newly infested berries were marked with a different color paint marker and counted. At 14-DAT, newly infested berries were counted and removed from each tagged branch and placed in a labeled container. Berries marked 7-DAT were also collected and held in a separate container. Lastly, berries marked on the DOT were counted, accumulated, and stored.

A minimum of 60 marked berries per treatment for DOT, 7-DAT, and 14-DAT were dissected to determine beetle positioning (A/B or C/D) and if alive, dead, or absent. If a CBB was found alive but had not yet entered the parchment and bean, positioning was considered A/B alive. An A/B dead CBB would not have entered the endosperm (bean) and may or may not have exhibited symptoms of *B. bassiana* infestation. An A/B absent CBB created an entrance hole, typically on the flower-end of the berry, but was not physically present in the hole. If a CBB entered the parchment and damaged the bean, positioning was considered C/D regardless of whether the beetle was dead, alive, or not located. Dissections were conducted as soon as possible and no later than 24 h after berry collection.

### 2.5. Statistical Analysis

The infestation rate data were analyzed by one-way analysis of variance (ANOVA) and means were separated by the Tukey–Kramer HSD test (JMP, Version 11. SAS Institute Inc., Cary, NC, USA, 1989–2019). The significance was calculated for the adult CBB positioning among treatments using a chi-squared test for independence (Microsoft Excel), conducted on the sample counts.

## 3. Results

CBB infestation rates among the three treatments were not different at the initiation of the experiment and remained similar throughout the trial (*p* > 0.05) (Table 1). No effect on new CBB populations infesting the berry was observed with any of the treatments.

CBB position in the berries was similar among treatments prior to the initial application (*p* > 0.05) (Table 2). Although all treatments exhibited lower percentages in the A/B alive position at 14-DAT, there were significant differences in whether the beetles were absent/dead (lower percentage for control) or moved to the C/D position. The implication is that the berries treated with spinetoram and *B. bassiana* had significantly fewer berries with damage to the coffee bean.

A closer examination of the results separated by when the berries were infested shows that the different treatments had effects over time (Table 3). At 14-DAT, the positions of CBBs in berries infested on DOT exhibit differences among treatments (*p* < 0.05) and are relative to the combined group in Table 1. The differences are less pronounced for the 7-DAT group (*p* = 0.07) and no differences were seen in berries infested between 7- and 14-DAT (*p* > 0.05). Berries infested on DOT demonstrated the greatest response to the different applications. There were 73% less live adult beetles in the A/B position in spinetoram-treated berries compared to berries with a water only treatment. The populations of live adult CBBs in the *B. bassiana* treatment were similar to the control. Berries treated with spinetoram and *B. bassiana* had 157% and 57% more beetles, respectively, that were dead or absent from the A/B position compared to the berries treated with water. As compared to the control, far fewer adults progressed to the C/D position with a 70% and 37% reduction in spinetoram and *B. bassiana* applications, respectively.

Berries infested 7-DAT showed more moderate differences among treatments with 14% and 34% reductions in live CBBs in the A/B position with spinetoram and *B. bassiana* applications, respectively. Populations of beetles in the A/B position that were dead or absent increased by 13% and 61% in the spinetoram and *B. bassiana*-treated berries, respectively, compared with the water control.

No differences were detected among treatments in berries newly infested between 7- and 14-DAT (*p* > 0.05).

## 4. Discussion

New infestations of CBBs were not reduced by applications of spinetoram in this study. Even *B. bassiana* treatments did not deter new infestations as seen in field trials where threshold-based applications of *B. bassiana* resulted in lower infestation rates [13]. This was not surprising since neither product is systemic with *B. bassiana*’s effectiveness that is dependent on direct contact with the insect’s cuticle or exoskeleton [3] and spinetoram causing a neurotoxic response with contact or ingestion [14]. In the current study, CBB continued to actively infest new berries on the tagged branches of each treatment. Since these plots were not isolated from neighboring branches; managed trees; and feral, unmanaged trees, CBBs were able to fly and crawl from one plot to another and from branch to branch.

Pre-treatment sampling of all three plots showed similar levels of infestation, bean damage, and CBBs in the A/B alive position. It appears there were initially low levels of a control agent, most likely native *B. bassiana*, as a small percentage of CBBs were dead and absent at the start of the trial. Differences were observed at the end of the trial, however, with berries treated with spinetoram and *B. bassiana* having significantly higher populations of dead and absent beetles than the water treatment. Fewer live adult beetles in the A/B position and less in the C/D position were also observed with spinetoram applications. In all infested berries at the end of the trial, *B. bassiana* had similar live adults in the A/B position but fewer beetles in the C/D position than untreated berries.

Spinetoram applications were most efficacious on berries infested prior to treatment, significantly reducing the number of live CBBs recovered from A/B and C/D positions compared to the control. Spinetoram also outperformed *B*. *bassiana* on berries infested on the DOT, although that trend was reversed with berries infested at 7-DAT. No differences between treatments were observed in 14-DAT-infested berries. These findings suggest that spinetoram may not have residual control of CBB 7-DAT, although *B. bassiana* may. This highlights the importance of applying spinetoram when CBBs are in the A/B position.

If CBBs are in the C/D position, the endosperm would be penetrated and bean damage would occur. Applications of spinetoram reduced CBB movement into the C/D position, thereby reducing damage to the bean and preventing CBB reproduction. Comparable to *B. bassiana* in its effectiveness against CBB, spinetoram might also be economically equivalent once the Hawaii Department of Agriculture *Beauveria* subsidy program concludes.

Due to concerns over the build-up of potential insect resistance to spinosyn products (group 5 insecticides), spinetoram should not be used as a complete replacement for *B. bassiana* for CBB management. The Delegate^®^ WG label recommends rotating to another class of insecticides after two applications of spinosyn-type products. Whereas multiple applications to control a single insect generation are acceptable, using the same insecticide group on consecutive generations should be avoided [14]. Spinosyn resistance is known to occur in several insect species including diamondback moth (*Plutella xylostella*) in Hawaii [15] and the Colorado potato beetle (*Leptinotarsa decemlineata*) [16]. Resistance management can be addressed by rotating spinetoram and *B. bassiana* applications, particularly in the beginning of the season when CBBs are in the A/B position.

Additional research needs to be conducted to investigate the short-term persistence of spinetoram since the lab results have indicated that there may be an indirect kill of CBBs following initial treatment [12]. The proper timing of applications depending on farm location should be investigated. Furthermore, spinetoram should be compared in field studies to pesticides containing pyrethrins with piperonyl butoxide (PY + PBO), which are currently used by some Hawaii coffee growers for CBB management. Costs per application vary by product and it is important to understand the cost–benefit of applying different combination control methods for CBB.

## 5. Conclusions

Spinetoram should be considered as an additional tool for CBB control as long as other IPM recommendations are followed. The application of spinetoram is most effective when beetles are in the A/B alive position and should be applied early in the coffee season to reduce CBB movement into the bean and prevent subsequent CBB reproduction.

## Figures and Tables

**Table 1:** Coffee berry borer (CBB) infestation on the day of treatment (DOT), 7 days after treatment (7-DAT), and 14 days after treatment (14-DAT) with spinetoram, *Beauveria bassiana*, or water.

	New CBB Infestation per Branch (%)		
Treatment	DOT	7-DAT	14-DAT
Spinetoram	11.5 ^a^	4.6 ^z^	2.3 ^A^
*B. bassiana*	9.9 ^a^	4.8 ^z^	2.5 ^A^
Water	10.4 ^a^	3.3 ^z^	2.6 ^A^

Means with the same letter within a column (different groups of letters for each column) are not statistically different according to the Tukey–Kramer HSD Test (*p* > 0.05). Chi-squared, Χ^2^ = 5.91 with 4 d.f., *p* = 0.21, computed on observations.

**Table 2:** Coffee berry borer (CBB) position in all infested berries on the day of treatment and 14 days after treatment with spinetoram, *Beauveria bassiana*, or water.

Day of Treatment			
	CBB Position in Berries (%)		
Treatment	A/B Alive	A/B Absent/Dead	C/D
Spinetoram	63.0	27.0	10.0
*B. bassiana*	69.5	18.9	11.6
Water	74.4	12.2	13.3
14 Days after Treatment			
	CBB Position in Berries (%)		
Treatment	A/B Alive	A/B Absent/Dead	C/D
Spinetoram	38.7	50.4	10.9
*B. bassiana*	44.9	46.9	8.3
Water	47.7	30.9	21.4

DOT: Chi-squared, Χ^2^ = 6.67 with 4 d.f., *p* = 0.15, computed on observations; 14-DAT: Chi-squared, Χ^2^ = 30.37 with 4 d.f., *p* < 0.005, computed on observations.

**Table 3:** Coffee berry borer (CBB) position 14 days after treatment with spinetoram, *Beauveria bassiana*, or water in berries infested on the day of treatment, and new berries infested 7 and 14 days after treatment.

Day of Treatment			
	CBB Position in Berries (%)		
Treatment	A/B Alive	A/B Absent/Dead	C/D
Spinetoram	6.7	80.0	13.3
*B. bassiana*	23.3	64.4	12.2
Water	24.4	31.1	44.4
7 Days after Treatment			
	CBB Position in Berries (%)		
Treatment	A/B Alive	A/B Absent/Dead	C/D
Spinetoram	54.9	34.1	11.0
*B. bassiana*	42.2	48.9	8.9
Water	63.6	30.3	6.1
14 Days after Treatment			
	CBB Position in Berries (%)		
Treatment	A/B Alive	A/B Absent/Dead	C/D
Spinetoram	62.1	30.3	7.6
*B. bassiana*	74.3	23.0	2.7
Water	64.1	31.3	4.7

DOT: Chi-squared, Χ^2^ = 54.84 with 4 d.f., *p* < 0.005, computed on observations; 7-DAT: Chi-squared, Χ^2^ = 8.52 with 4 d.f., *p* = 0.07, computed on observations; 14-DAT: Chi-squared, Χ^2^ = 3.64 with 4 d.f., *p* = 0.46, computed on observations.

## Data Availability

The data presented in this study are available on request from the corresponding author.
